# Deutscher Arzneimittel-Codex-aligned multi-dimensional quality assessment of Gastrodiae Rhizoma granules

**DOI:** 10.3389/fphar.2026.1843493

**Published:** 2026-05-15

**Authors:** Yin Xiong, Yiwei Wu, Xiaoyan Che, Xiuming Cui, Ryan Gene Hornbeck, Yuefei Geng, Ruoying Wu, Jin Tan, Yindi Zhu

**Affiliations:** 1 School of Chinese Medicine, Wenzhou Medical University, Wenzhou, China; 2 Leiden University-European Center for Chinese Medicine and Natural Compounds, Leiden, Netherlands; 3 Kunming University of Science and Technology, Kunming, China; 4 Wenzhou-Kean University, Wenzhou, China; 5 Good Doctor Pharmaceutical Group Co., Ltd., Chengdu, China

**Keywords:** Deutscher Arzneimittel-Codex, Gastrodiae Rhizoma granules, quality assessment, quantitative analysis of multi-components by single marker, system suitability test

## Abstract

**Background:**

Reproducible quality assessment is essential for the standardization and international comparability of herbal granule products. Gastrodiae Rhizoma granules (GRG) are widely used herbal preparations, yet analytical strategies aligned with different compendial requirements remain limited. The study aimed to establish and evaluate a Deutscher Arzneimittel-Codex (DAC)-aligned analytical strategy for the quality assessment of GRG and compare it with the Chinese national standard.

**Methods:**

A multi-dimensional analytical approach integrating thin-layer chromatography (TLC), quantitative analysis of multi-components by single marker (QAMS), and high-performance liquid chromatography (HPLC) fingerprinting was developed for GRG. TLC was assessed using DAC system suitability requirements. Gastrodin was used as the internal reference for simultaneous determination of *p*-hydroxybenzyl alcohol and parishins E, B, C, and A by QAMS. HPLC fingerprints were established for 15 batches of GRG and evaluated by cosine similarity analysis, principal component analysis, hierarchical clustering analysis, and radar plot visualization.

**Results:**

The TLC method achieved clear separation of gastrodin and parishin B, satisfying DAC suitability requirements. QAMS results showed no significant difference (*P* > 0.05) compared with the external standard method, demonstrating good accuracy and reliability. HPLC fingerprint analysis revealed generally consistent chemical profiles among the investigated batches, while also identifying inter-batch variation in several samples. Chemometric analyses further supported the overall similarity and localized heterogeneity of the investigated batches. Compared with the Chinese national standard, the DAC-aligned strategy placed greater emphasis on chromatographic suitability and multi-component characterization.

**Conclusion:**

This study demonstrates the feasibility of a DAC-aligned analytical strategy for the multi-dimensional quality assessment of GRG, and offers a useful reference for cross-standard comparison and for the harmonization of quality evaluation approaches of herbal granule products.

## 1 Introduction

Traditional Chinese medicine (TCM) granules are modern herbal preparations derived from decoction pieces through standardized manufacturing processes, including extraction, concentration, separation, drying, granulation, and packaging (Cui et al., 2022; Gu et al., 2025). Compared with traditional decoctions, these granules offer advantages such as ease of storage and transport, more accurate dosing, improved patient compliance, and more consistent quality control (Zhao et al., 2024). As a result, TCM granules have been increasingly used in clinical practice and have attracted growing attention in international markets.

Despite this development, the quality evaluation of TCM granules remains challenged by differences in regulatory frameworks and technical requirements across countries and regions. In particular, variation in quality indices, chromatographic requirements, and validation criteria has created practical obstacles for the international registration and broader acceptance of herbal medicinal products. The Deutscher Arzneimittel-Codex (DAC), an important compendial and technical reference in Germany, is used for the quality evaluation of medicinal products and pharmaceutical starting materials. In the context of herbal analysis, DAC-oriented requirements are broadly consistent with European Pharmacopoeia (EP)-style quality expectations and place particular emphasis on chromatographic suitability, defined reference substances, and batch-to-batch consistency evaluation (Hempen, 2014; Manns et al., 2019). Therefore, these requirements can impose stricter demands on the characterization and quality assessment of Chinese herbal granule products intended for international use.

Gastrodiae Rhizoma (GR, 天麻 in Chinese), the dried tuber of *Gastrodia elata* Blume, is a well-recognized herbal medicine that has long been used in Chinese medicine for conditions such as headache, dizziness, and convulsive disorders (Chinese Pharmacopoeia Commission, 2025; Hsu et al., 2021). Phytochemical and pharmacological studies have demonstrated that its major bioactive constituents, such as gastrodin, *p*-hydroxybenzyl alcohol, and parishins, are commonly regarded as important marker components for its quality evaluation (Ma et al., 2024; Su et al., 2023). To meet modern clinical needs, GR has been developed into a TCM granule form and included in the Chinese national standard (CNS, YBZ-PFKL-2021122) as a dosage form used in place of traditional decoction pieces (National Medical Products Administration, 2021). Although the current CNS provides a basic framework for quality control, differences remain between the CNS and DAC-oriented requirements in areas such as system suitability criteria, reference standard use, and the overall depth of chromatographic characterization. These differences complicate the cross-standard evaluation of GR granules (GRG), particularly in the context of international quality assessment.

In this context, the present study aims to develop and evaluate a DAC-aligned analytical framework for the quality assessment of GRG. Here, “DAC-aligned” refers to the adoption of analytical elements consistent with DAC/EP-style expectations, rather than direct implementation of a legally binding registration standard for GRG. The strategy integrated thin-layer chromatography (TLC) for characteristic component identification, quantitative analysis of multi-components by a single marker (QAMS) for simultaneous quantification of marker components, and high-performance liquid chromatography (HPLC) fingerprinting for chemical consistency evaluation. The proposed approach was further examined in comparison with the current CNS in terms of analytical requirements, quality indicators, and methodological characteristics. This work provides a practical reference for cross-standard comparison and for the harmonization of quality evaluation approaches for herbal granule products.

## 2 Materials and methods

### 2.1 GRG samples

Fifteen batches of commercial GRG from different manufacturers in China were collected for analysis. GRG is a granule preparation derived from Gastrodiae Rhizoma (Tianma; the dried tuber of *G. elata* Blume, Orchidaceae). All samples analyzed in this study were marketed products with identifiable batch numbers and manufacturer information. According to the CNS, GRG are prepared from GR decoction pieces by decoction, concentration, drying, and granulation, with addition of excipients as needed. Based on the reported dry extract yield of 14%–25%, the native drug-extract ratio (DER native) is approximately 4.0–7.1:1. According to information provided by the manufacturers, maltodextrin is commonly used as an excipient when excipients are included. Detailed information on the samples is provided in Table 1.

**TABLE 1:** Information on the GRG samples analyzed in the study.

Sample no.	Batch no.	Manufacturer
S1	1039192	Guangdong Yifang Pharmaceutical Co., Ltd.
S2	1042132	Guangdong Yifang Pharmaceutical Co., Ltd.
S3	1052223	Guangdong Yifang Pharmaceutical Co., Ltd.
S4	1024412	Guangdong Yifang Pharmaceutical Co., Ltd.
S5	2101001W	China Resources Sanjiu Medical & Pharmaceutical Co., Ltd.
S6	19045512	Beijing Tcmages Pharmaceutical Co., Ltd.
S7	21060781	Tianjiang Pharmaceutical Co., Ltd.
S8	0129181	Guangdong Yifang Pharmaceutical Co., Ltd.
S9	0079081	Guangdong Yifang Pharmaceutical Co., Ltd.
S10	1052221	Guangdong Yifang Pharmaceutical Co., Ltd.
S11	0129171	Guangdong Yifang Pharmaceutical Co., Ltd.
S12	0079101	Guangdong Yifang Pharmaceutical Co., Ltd.
S13	0079091	Guangdong Yifang Pharmaceutical Co., Ltd.
S14	20041371	Tianjiang Pharmaceutical Co., Ltd.
S15	0049231	Guangdong Yifang Pharmaceutical Co., Ltd.

### 2.2 Chemicals

The reference standards of gastrodin (no. 110807-202010, purity HPLC ≥ 95.5%) and *p*-hydroxybenzyl alcohol (no. 11970-201702, purity HPLC ≥ 99.4%), and the herb reference substance (HRS) of GR (no. 120944-202112) were purchased from National Institutes for Food and Drug Control (Beijing, China). Parishin A (no. PRF20091710, purity HPLC ≥ 98%), parishin B (no. PRF21030901, purity HPLC ≥ 98%), and parishin E (no. PRF20092841, purity HPLC ≥ 98%) were purchased from Biopurify Phytochemicals Ltd. (Chengdu, China). Parishin C (no. D06N11S130122, purity HPLC ≥ 98%) was purchased from Shanghai Yuanye Biotechnology Co., Ltd. (Shanghai, China). Acetonitrile was of HPLC grade. All other reagents were of analytical grade.

### 2.3 TLC identification

#### 2.3.1 Preparation of the test solution

A total of 0.1 g of GRG was accurately weighed and extracted with 10 mL of methanol by ultrasonic extraction at 200 W and 53 kHz for 30 min. After filtration, the filtrate was evaporated to dryness, and the residue was redissolved in 0.5 mL of methanol to obtain the test solution.

#### 2.3.2 Preparation of the HRS solutions

GR herbal reference standard (HRS) of 0.3 g was extracted with 8.0 mL of water under reflux for 2 h. The extract was filtered and the filtrate was evaporated to dryness. The residue was dissolved in 10.0 mL of methanol and subjected to ultrasonic extraction for 30 min. After filtration, the filtrate was again evaporated to dryness, and the final residue was dissolved in 0.5 mL of methanol to obtain the HRS solution.

#### 2.3.3 Preparation of reference standard solutions

Reference solution a, for SST, was prepared by dissolving 2.0 mg of gastrodin and 2.0 mg of parishin B in 1.0 mL of methanol to produce a mixed reference solution.

Reference solution b was prepared by dissolving 2.0 mg of gastrodin in 1.0 mL of methanol.

Reference solution c was obtained by diluting 0.25 mL of Reference solution b to 1.0 mL with methanol to obtain a concentration of 0.5 mg/mL.

#### 2.3.4 Chromatographic conditions

TLC was performed using a silica gel plate (Macherey-Nagel, 5 × 10 cm, particle size 5–17 μm). The mobile phase consisted of methylene chloride, ethyl acetate, methanol, and water in the ratio of 2:4:2.5:1 (*V/V/V/V*). Aliquots of 4 μL of each solution were applied as 8 mm bands onto the same TLC plate. Chromatographic development was carried out in an unsaturated chamber to a distance of 8 cm. The developed plate was dried in air and sprayed with a 10% (*V/V*) sulfuric acid solution in ethanol, followed by heating at 105 °C for 15 min. The chromatographic profiles were examined under daylight and under UV light at 310 nm. Observation at 310 nm was selected because it provided the clearest visualization of the characteristic bands under the present conditions. In the chromatogram of the test solution, spots corresponding in position and color to those of the HRS and reference solutions were observed. The SST of the TLC system was assessed using Reference solution a.

### 2.4 HPLC analysis

#### 2.4.1 Preparation of the test solution

GRG sample of 0.2 g was accurately weighed and extracted with 25.0 mL of a methanol–water solvent mixture (30:70, *V/V*). The mixture was sonicated at 200 W and 53 kHz for 30 min, then cooled to room temperature. The solution was weighed again, and any lost volume was compensated with the same solvent mixture. The mixture was shaken thoroughly, allowed to stand, and then filtered through a 0.22 μm membrane filter. The resulting filtrate was used as the test solution.

#### 2.4.2 Preparation of reference standard solutions

Reference solution a, for SST, was prepared by dissolving 0.1 mg each of parishin B and parishin C in 10.0 mL of the same solvent mixture.

Reference solution b was prepared by dissolving 2.0 mg of gastrodin in 10.0 mL of the methanol–water (30:70, *V/V*) solvent mixture.

Reference solution c was prepared by dissolving 2.0 mg of gastrodin, 0.06 mg of *p*-hydroxybenzyl alcohol, 0.45 mg of parishin E, 0.13 mg of parishin B, 0.1 mg of parishin C, and 0.1 mg of parishin A in 10 mL of the methanol–water (30:70, *V/V*) solvent mixture.

#### 2.4.3 Chromatographic conditions

The analysis was performed using a HPLC system equipped with a reversed-phase C18-AQ column (250 mm × 4.6 mm, 5 μm particle size). The mobile phase consisted of: mobile phase A of 0.1% phosphoric acid aqueous solution (*V/V*), and mobile phase B of acetonitrile. The gradient mode was as follows: 2%–6% B in 0–16 min; 6%–12% B in 16–22 min; 12%–18% B in 22–35 min; 18%–24% B in 35–42 min; 24%–95% B in 42–43 min. The flow rate was set at 1.0 mL/min. The detection wavelength was 220 nm. The injection volume was 5 µL.

#### 2.4.4 Method validation

##### 2.4.4.1 Specificity

The specificity of the method was evaluated by comparing the chromatograms of the blank solvent, the test solution, reference solution a and reference solution c. Chromatographic conditions were identical to those used in Section 2.4.3. Peaks in the test solution were identified by comparing their retention times with those of the reference substances, and no interference was observed from the blank solution.

##### 2.4.4.2 Precision

###### 2.4.4.2.1 Instrument precision

Instrument precision was assessed by injecting reference solution c for six consecutive times. The relative standard deviation (RSD) of the peak area for each analyte was calculated to evaluate the repeatability of the chromatographic system.

###### 2.4.4.2.2 Repeatability

Repeatability was examined by preparing and analyzing six independently prepared test solutions from the **s**ame batch of GRG (S9). The RSDs of the peak areas were calculated to assess intra-batch consistency.

###### 2.4.4.2.3 Intermediate precision

Intermediate precision was evaluated by analyzing twelve test solutions prepared from the same batch (S9). The analyses were performed on different days, by different analysts, and using different chromatographic instruments. The RSD values of the peak areas were calculated to assess method robustness under variable laboratory conditions.

##### 2.4.4.3 Stability

The stability of the sample solution was assessed by analyzing a test solution prepared from batch S9 at different time points: 0 h, 4 h, 8 h, 18 h, 24 h, and 36 h. The results were used to evaluate the solution’s short-term stability under laboratory conditions.

##### 2.4.4.4 Accuracy

Accuracy was determined by a recovery experiment using batch S9 of GRG. A total of three groups of spiked samples were prepared, each group containing three replicates. For each sample, 0.125 g of GRG powder was accurately weighed, and known amounts of six reference standards (gastrodin, *p*-hydroxybenzyl alcohol, parishin E, parishin B, parishin C, and parishin A) were added. The recovery percentages were calculated to evaluate the accuracy of the method.

##### 2.4.4.5 Linearity, range, limit of detection (LOD), and limit of quantification (LOQ)

Linearity was evaluated by preparing and analyzing six different concentrations of a mixed reference standard solution containing gastrodin, *p*-hydroxybenzyl alcohol, parishin E, parishin B, parishin C, and parishin A. Calibration curves were constructed for each compound, and correlation coefficients (*R* ^2^) were calculated to assess linearity within the tested concentration range. The sensitivity was evaluated by LOD and LOQ.

#### 2.4.5 Development of QAMS

##### 2.4.5.1 Determination of RCF

The QAMS method was developed according to available methodology with minor modifications (Li et al., 2019). Gastrodin, p-hydroxybenzyl alcohol, and parishin-type compounds were selected as target analytes because they are characteristic constituents of Gastrodiae Rhizoma and are relevant to its chromatographic characterization, quality evaluation, and clinical efficacies. Different compounds were evaluated as candidate internal references. The final internal reference (IR) and the corresponding relative correction factors (RCFs) were selected based on low RSD values, stable chromatographic behavior, and suitable physicochemical properties.

The RCFs were calculated using the calibration curves as follows:

$$F_{i/s}=\frac{F_{i}}{F_{s}}=\frac{A_{i}/C_{i}}{A_{s}/C_{s}}=\frac{A_{i}\times C_{s}}{A_{s}\times C_{i}}$$ (1)

where $F_{i/s}$ is the RCF; $C_{i}$ and $C_{s}$ represent the concentrations of the analyte and internal reference, respectively; and $A_{i}$ and $A_{s}$ represent their corresponding peak areas.

The external standard method (ESM) and QAMS were employed to quantify six components in each sample, and the results were compared using RSD as error indices to verify the feasibility of the QAMS method.

$F_{i/s}$ was used to determine the concentrations of individual component ( $C_{i}$ ) as follows:

$$C_{i}=\frac{A_{i}\times C_{s}}{A_{s}\times F_{i/s}}$$ (2)

##### 2.4.5.2 Quantification of six components in GRG

The percentage content of gastrodin ( $W_{i}$ ) was calculated as follows:

$$W_{i}=\frac{C_{i}\times V}{M_{s}}\times 100\%$$ (3)

where V is the extract volume (mL) and $M_{s}$ is the mass of sample (mg).

#### 2.4.6 HPLC fingerprinting analysis

##### 2.4.6.1 Establishment of HPLC fingerprints and similarity evaluation

HPLC fingerprinting was performed to evaluate the overall chemical profile and batch-to-batch consistency of commercial GRG samples. Chromatographic peak alignment was carried out using a retention time (RT) tolerance of ±0.2 min to correct for minor variations between batches. A total of 58 aligned peaks were identified in the retention time range of 0–43 min for fingerprint similarity analysis, while a subset of selected characteristic peaks was used for visualization and chemometric interpretation.

A fingerprint profile was established for each batch based on the aligned peak areas. The similarity between individual batches was quantitatively evaluated using cosine similarity coefficients, with values closer to 1.00 indicating greater similarity. A similarity heatmap was constructed to visualize the correlation among batches. In addition, a heatmap of peak area distributions was generated to display the relative abundance of each compound across all batches, facilitating intuitive assessment of batch-to-batch variation and identification of outliers.

##### 2.4.6.2 Chemometric analysis

To evaluate chemical consistency and support batch classification, multivariate chemometric methods were applied to the aligned fingerprint data matrix:

Principal component analysis (PCA) was performed to reduce dimensionality and visualize sample clustering (Greenacre et al., 2022). The first two principal components (PC1 and PC2) were plotted to show groupings or deviations among the 15 batches.

Hierarchical Cluster Analysis (HCA) was conducted using Euclidean distance and Ward’s method to construct a dendrogram, classifying batches based on chemical profile similarity (Essary et al., 2022).

Radar plots were used to visualize the normalized peak distributions of selected characteristic constituents (Seide et al., 2021). Radar charts were constructed using the seven characteristic peaks (RT = 12.88, 14.68, 18.12, 29.28, 33.76, 35.20, and 40.83 min). All radar plots were normalized by maximum peak area within each batch to reflect relative compound distribution, enabling fingerprint pattern comparison independent of total content.

### 2.5 Statistical analysis

Data processing and visualization were performed using GraphPad Prism 9.3.1 (GraphPad Software, San Diego, CA, United States), IBM SPSS Statistics 26 (IBM North America, New York, NY, United States), and Python 3.11 (Python Software Foundation). RSD, Pearson correlation, cosine similarity, and resolution values were calculated for method validation and chemometric evaluation. Multivariate data analyses were conducted using standardized peak area matrices, and graphical outputs were generated for inclusion in the manuscript.

## 3 Results

### 3.1 TLC identification

#### 3.1.1 TLC SST

**FIGURE 1:**
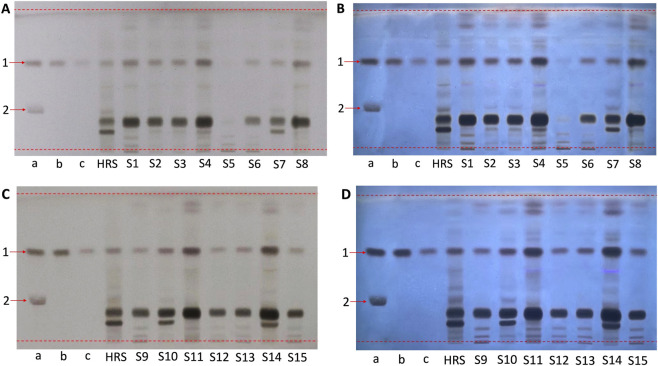
TLC identification results of GRG samples **(A)** S1–S8 and **(C)** S9–S15 in daylight, and **(B)** S1–S8 and **(D)** S9–S15 under ultraviolet light of 310 nm. a reference solution a, b. Reference solution b, c. Reference solution c. 1. Gastrodin, 2. Parishin B.

SST was performed using a mixed reference solution containing both gastrodin and parishin B (Figure 1). The Rf values of the two compounds were approximately 0.60 and 0.27, respectively, and the two bands were clearly separated under the established chromatographic conditions.

#### 3.1.2 Identification of GRG samples

Gastrodin was used as the principal marker for TLC identification, and two concentrations of the gastrodin reference solution were prepared. As shown in Figure 1, the reference solution and its 1/4-dilution produced clearly distinct band intensities, and the GR HRS exhibited well-resolved characteristic bands. All investigated samples exhibited a band corresponding in position to the gastrodin reference band, while the overall major band pattern of most samples was consistent with that of the GR HRS.

Some inter-batch and inter-manufacturer differences were also observed. Most samples from Guangdong Yifang Pharmaceutical Co. exhibited generally similar band patterns, whereas samples from other manufacturers (e.g., S5–S7 and S14) showed minor variations in the intensity or distribution of secondary bands. Batch S5 showed a comparatively weaker TLC response than most other samples.

### 3.2 HPLC analysis

#### 3.2.1 HPLC SST

Under the established chromatographic conditions, the resolution between parishin B and parishin C exceeded 4.5, which was substantially higher than the minimum requirement of 1.5 specified in the EP/DAC guidelines.

#### 3.2.2 Method validation

##### 3.2.2.1 Specificity

**FIGURE 2:**
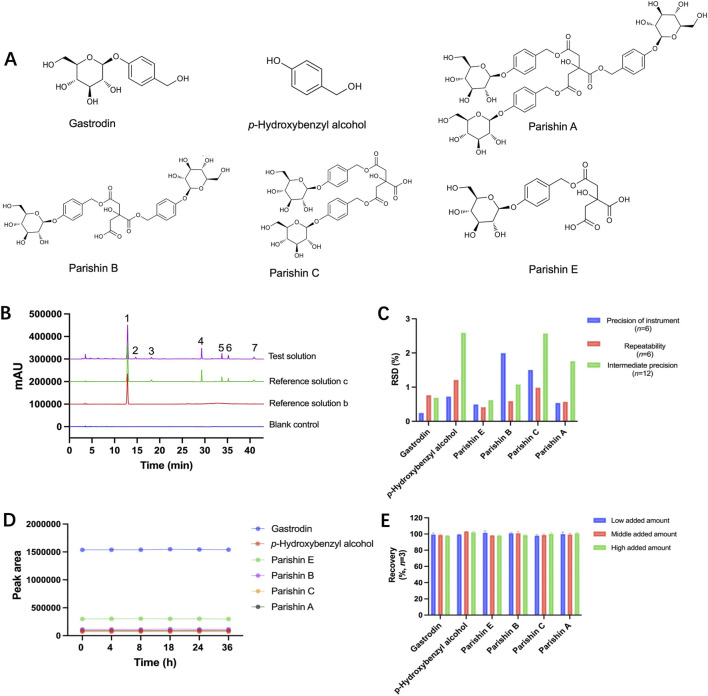
**(A)** Structures of six marker compounds, **(B)** HPLC chromatogram of the blank control, reference solutions and test solution, **(C)** RSD values of precision of instrument, repeatability, and intermediate precision, **(D)** stability of 6 marker compounds across 36 h, and **(E)** recovery of 6 marker compounds. 1. Gastrodin, 2. Unknown constituent, 3. *p*-hydroxybenzyl alcohol, 4. Parishin E, 5. Parishin B, 6. Parishin C, 7. Parishin A.

The reference solution with six reference compounds (Figure 2A) was prepared for the specificity test. The results in Figure 2B showed that no interfering peaks were observed in the chromatogram of the blank control solution. All six marker compounds in reference solution c produced distinct and well-defined peaks at their respective retention times. Corresponding peaks were also detected in the test solution at the same positions, with satisfactory peak shapes and no abnormal impurity signals observed.

##### 3.2.2.2 Precision

###### 3.2.2.2.1 Precision of instrument

The reference solution c was consecutively injected for six times to assess the instrument precision. The RSDs of gastrodin, *p*-hydroxybenzyl alcohol, parishin E, parishin B, parishin C, and parishin A were 0.24%, 0.73%, 0.50%, 2.00%, 1.50%, and 0.54% (*n* = 6), respectively (Figure 2C).

###### 3.2.2.2.2 Repeatability

Six test solutions from batch S9 were independently prepared for the repeatability test. The RSDs of peak areas of gastrodin, *p*-hydroxybenzyl alcohol, parishin E, parishin B, parishin C, and parishin A were 0.76%, 1.21%, 0.42%, 0.59%, 0.98%, and 0.58% (*n* = 6), respectively (Figure 2D).

###### 3.2.2.2.3 Intermediate precision

Twelve test solutions from batch S9 were prepared and determined by different technicians and instruments on different dates. The corresponding RSDs for gastrodin, p-hydroxybenzyl alcohol, parishin E, parishin B, parishin C, and parishin A were 0.69%, 2.59%, 0.62%, 1.08%, 2.57%, and 1.76% (n = 12), respectively (Figure 2C).

##### 3.2.2.3 Stability

One test solution prepared from batch S9 was determined at the 0 h, 4 h, 8 h, 18 h, 24 h, and 36 h, respectively. The RSDs of peak areas of gastrodin, p-hydroxybenzyl alcohol, parishin E, parishin B, parishin C, and parishin A were 0.27%, 0.80%, 0.54%, 2.19%, 1.65%, and 0.59% (n = 6), respectively (Figure 2D).

##### 3.2.2.4 Accuracy

Accuracy was assessed by performing a recovery experiment using batch S9. Three groups of spiked samples were prepared, with three replicates in each group. Known amounts of gastrodin, p-hydroxybenzyl alcohol, parishin E, parishin B, parishin C, and parishin A were added into the samples prior to analysis. As shown in Figure 2E, the recoveries of the six analytes were within an acceptable range.

##### 3.2.2.5 Linearity, range, LOD, and LOQ

Six concentrations of the mixed reference solution of gastrodin, p-hydroxybenzyl alcohol, parishin E, parishin B, parishin C, and parishin A were prepared and determined. The regression equations, linear ranges, LODs, and LOQs of the six compounds were shown in Table 2. The results showed that the linearity of each compound was good in the corresponding range.

**TABLE 2:** Calibration curves, linear ranges, LODs, and LOQs of the six reference compounds (*n* = 6).

Compound	Regression equation	*R* ^*2*^	Linear range (mg/mL)	LOD (mg/mL)	LOQ (mg/mL)
Gastrodin	*y* = 6744078*x* + 24495	0.9995	0.02∼0.6	0.0163	0.0494
*p*-Hydroxybenzyl alcohol	*y* = 12307694*x* + 679.5	0.9997	0.0006∼0.018	0.0004	0.0012
Parishin E	*y* = 6060054*x* + 2441	0.9996	0.0045∼0.135	0.0033	0.0099
Parishin B	*y* = 8001789*x* – 672.8	0.9998	0.0013∼0.039	0.0006	0.0019
Parishin C	*y* = 8268232*x* – 554.8	0.9995	0.001∼0.03	0.0008	0.0026
Parishin A	*y* = 8728848*x* + 1,209	0.9998	0.001∼0.03	0.0006	0.0017

#### 3.2.3 Development of QAMS

##### 3.2.3.1 Determination of RCFs

**FIGURE 3:**
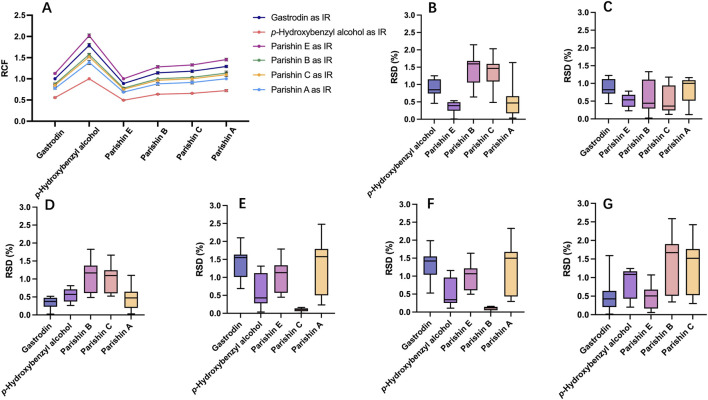
**(A)** RCFs of six components with each of them as IR and RSDs between QAMS and ESM using **(B)** gastrodin, **(C)** *p*-hydroxybenzyl alcohol, **(D)** parishin E, **(E)** parishin B, **(F)** parishin C, and **(G)** parishin A as IR.

Seven concentrations of components were prepared to calculate RCFs according to Equation 1 for different IRs. As shown in Figure 3A, the RSDs of the RCFs ranged from 1.30% to 2.92%, 1.51%–3.66%, 1.30%–2.50%, 1.05%–4.09%, 1.04%–3.92%, and 2.24%–4.22% when gastrodin, *p*-hydroxybenzyl alcohol, parishin E, parishin B, parishin C, and parishin A were used as IRs, respectively.

Combined with the comparison results of ESM and QAMS using different components as IRs (Figures 3B–G), it was observed that the difference between the two methods was not significant. Gastrodin chosen as the final IR for subsequent QAMS analysis. Using grastrodin as the IR, the mean RCFs of *p*-hydroxybenzyl alcohol, parishin E, parishin B, parishin C, and parishin A obtained as 0.5581, 1.1259, 0.8781, 0.8481, and 0.7739, respectively. For these five components, the RCF deviations were all below 3%.

##### 3.2.3.2 Quantification of six components in GRG

**FIGURE 4:**
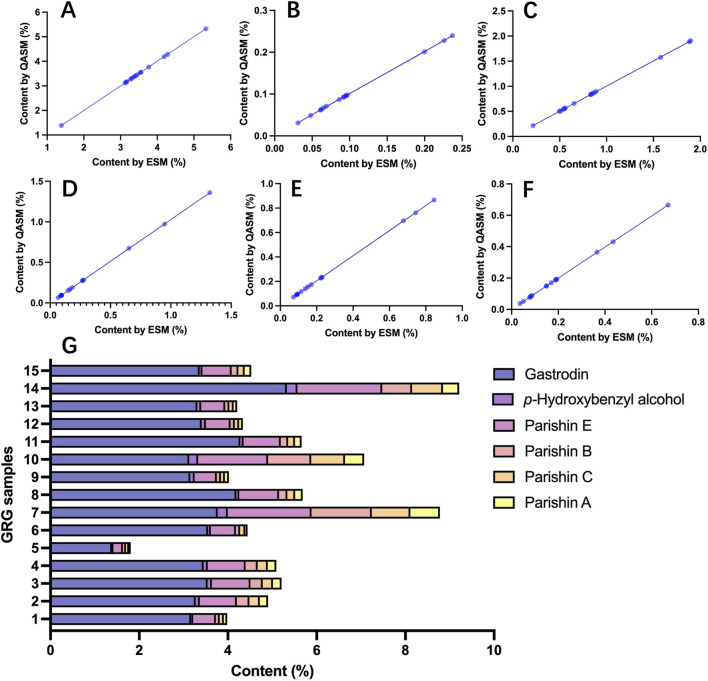
**(A–F)** Correlation scatter plots comparing ESM and QAMS content values and **(G)** contents of six components in GRG samples.

The contents of gastrodin, p-hydroxybenzyl alcohol, parishin E, parishin B, parishin C, and parishin A in 15 batches of sample were determined by both ESM and QAMS (Equations 2, 3). According to the correlation scatter plots (Figures 4A–F), the points were closely aligned with the regression line, with *R* ^2^ = 0.999 or 1.000.

Based on the established QAMS method, the six target components were quantified in the investigated GRG batches (Figure 4G). Gastrodin was the predominant constituent, with contents ranging from 1.381% to 5.312%. The remaining components followed a descending order of average content: parishin E (0.504%–1.902%), parishin B (0.066%–1.348%), parishin C (0.071%–0.859%), and parishin A (0.037%–0.660%). The content of *p*-hydroxybenzyl alcohol was relatively low and was below 0.1% in most samples. When the six components were considered together, 14 of the 15 investigated batches exhibited total contents above 3.5%.

#### 3.2.4 HPLC fingerprinting analysis

##### 3.2.4.1 Establishment of HPLC fingerprints and similarity evaluation

**FIGURE 5:**
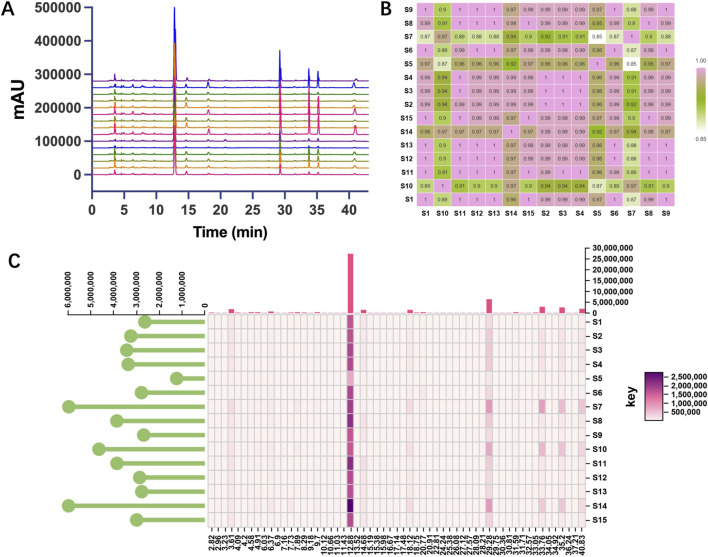
**(A)** The characteristic HPLC fingerprints of 15 batches of GRG samples, **(B)** cosine similarity coefficients of all pairwise batch comparisons, and **(C)** heatmap of peak areas of all detected peaks across batches.

HPLC fingerprints of 15 batches of GRG were established under the optimized gradient elution conditions (Figure 5A). Peak alignment was performed with a retention time (RT) tolerance of ±0.2 min to correct for minor shifts across batches. A total of 58 aligned peaks were identified within the RT range of 0–43 min. These peaks served as the basis for comparative evaluation of the overall chemical profiles of the investigated batches. Among them, seven major peaks with relatively stable retention behavior, good peak shape, and consistent occurrence across batches were selected as characteristic peaks of GRG fingerprints, with RT at 12.88, 14.68, 18.12, 29.28, 33.76, 35.20, and 40.83 min, respectively. By comparison with the mixed reference standard solution (Figure 2B), six components corresponding to the major peaks were identified as gastrodin (RT = 12.88 min), *p*-hydroxybenzyl alcohol (RT = 18.12 min), parishin E (RT = 29.28 min), parishin B (RT = 33.76 min), parishin C (RT = 35.20 min), and parishin A (RT = 40.83 min), while one peak remained unidentified.

To assess fingerprint similarity, cosine similarity coefficients were calculated for all pairwise batch comparisons among the 15 batches (Figure 5B). Most batches exhibited similarity values greater than 0.95. Notably, S7 and S10 showed lower similarity values compared to the rest, with values falling below 0.95 against several other batches. S5 showed the lowest similarity to S7.

A heatmap of aligned peak areas was further produced to display the abundance distribution of all detected peaks across batches (Figure 5C). Most samples showed broadly similar intensity distributions for the majority of peaks. S7, S10, and S14 showed relatively stronger responses in some later-eluting peaks, whereas S5 showed generally weaker signals across a broad range of the fingerprint.

##### 3.2.4.2 Chemometric analysis

**FIGURE 6:**
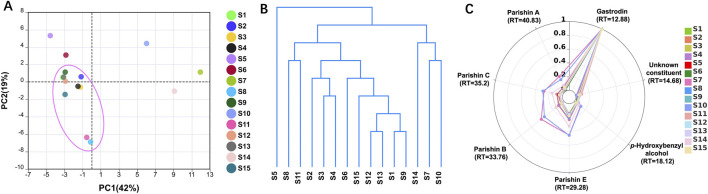
Chemometrical analyses of **(A)** PCA score plot, **(B)** HCA dendrogram, and **(C)** radar plot of 15 GRG samples.

To further explore batch-to-batch variation and sample clustering, PCA and HCA were performed on the aligned peak area matrix. As shown in the PCA score plot (Figure 6A), the first two principal components accounted for 42% and 19% of the total variance, respectively. Most batches were clustered tightly, whereas S5, S7, S10, and S14 were separated from the main cluster.

The HCA dendrogram showed a clustering pattern broadly consistent with the PCA results (Figure 6B). S5 was positioned on the distinct branch, consistent with its lower similarity coefficients and generally reduced peak intensity. S7, S8, and S10, which showed higher signal intensities for multiple peaks in Figure 5C, were grouped into a closely related subcluster.

To visualize the relative distribution of selected characteristic constituents, a radar plot were constructed using seven characteristic peaks at 12.88, 14.68, 18.12, 29.28, 33.76, 35.20, and 40.83 min, corresponding to gastrodin, an unidentified constituent, *p*-hydroxybenzyl alcohol, and parishins E, B, C, and A, respectively (Figure 6C). All data were normalized per batch to reflect the relative distribution of components. Gastrodin was highlighted as the dominant component across all batches in the plot, whereas notable inter-batch variation was observed for the remaining constituents. S5 and a few others demonstrated compressed and narrower radar shapes. In contrast, S7 and S14 displayed well-rounded and expansive patterns in the later-eluting region.

## 4 Discussion

### 4.1 DAC-aligned TLC SST and identification for GRG

In the present study, “DAC-aligned” refers to the incorporation of analytical elements consistent with DAC/EP-style expectations, including explicit system suitability testing in TLC and HPLC, graded TLC reference solutions, and integrated multi-component and multi-batch characterization. For the design of the TLC identification method, the requirements in the EP (European Pharmacopoeia Commission, 2022) and DAC present differences from those in the Chinese Pharmacopoeia (ChP) (Chinese Pharmacopoeia Commission, 2025). In the EP/DAC framework, SST places emphasis on the inclusion of two substances with similar retention factor (Rf) values as SST markers—regardless of whether they are present in the test sample—to assess separation under the chromatographic conditions. In contrast, the ChP approach places less emphasis on such paired-marker SST and instead focuses mainly on the acceptable distribution of spots or bands within a defined Rf range. In the study, the SST results showed that gastrodin and parishin B were clearly separated under the established chromatographic conditions, fulfilling the relevant EP/DAC suitability expectations for chromatographic separation, and indicating that the TLC system was appropriate for subsequent qualitative identification of GRG samples.

In addition to SST, differences were also observed between the Chinese and European approaches to reference solution design. The current ChP and CNS for GRG mainly rely on a single-concentration reference solution for spot comparison, whereas the EP/DAC-oriented approach places greater emphasis on the use of graded reference solutions to assess both signal intensity and chromatographic performance across concentration levels. For herbal granule preparations, comparison with an extract or herbal reference substance of the source crude drug is also important for evaluating whether the characteristic chromatographic features of the granule are consistent with those of the declared herbal material (European Pharmacopoeia Commission, 2022). In the study, the clearly distinct band intensities of two concentrations of gastrodin reference solution indicated that the optimized TLC conditions were suitable for qualitative assessment of GRG (Figure 1). The similar band pattern of investigated samples with that of the GR HRS also indicate that the analyzed granules were broadly consistent with the declared herbal source. Some inter-batch and inter-manufacturer variation may reflect differences in raw material quality, extraction process, or manufacturing conditions, although the specific cause cannot be determined from TLC analysis alone.

Overall, the TLC results demonstrated that the developed method was suitable for rapid qualitative identification of GRG and for assessing basic consistency with the herbal reference material. However, as TLC provides primarily qualitative information and limited chemical resolution, it is insufficient for detailed evaluation of batch-to-batch compositional variation. Therefore, HPLC-based quantitative analysis and fingerprint profiling were further performed to characterize the chemical composition of GRG more comprehensively.

### 4.2 DAC-aligned HPLC SST and chromatographic conditions for GRG

In contrast to the ultra-high-performance liquid chromatography system adopted in the current CNS for GRG, this study employed a conventional HPLC system to establish the fingerprinting and quantitative analysis. This choice was intended to improve the practicality and transferability of the method, as conventional HPLC systems remain widely used in pharmacopoeial and quality-control laboratories. From the perspective of cross-standard application, such an approach may facilitate broader implementation and inter-laboratory reproducibility.

In the EP/DAC framework, SST commonly requires the use of a reference solution containing the target analyte and a structurally related neighboring compound. When no reference substance is available for the adjacent peak, the SST is evaluated using the resolution between the target compound and its neighboring peak in the test solution. Importantly, the adjacent peak used for SST evaluation must have a known chemical structure; the resolution between a target compound and an unknown peak is not considered acceptable (European Pharmacopoeia Commission, 2022). This requirement is particularly relevant for herbal preparations, in which complex matrices may otherwise compromise the reliability of chromatographic interpretation. In this study, the high resolution between parishin B and parishin C under the established chromatographic conditions indicated that the system provided adequate separation for subsequent analysis.

The method validation results in Figure 2 also demonstrated that the established HPLC method possessed adequate specificity, precision, stability, accuracy, linearity, and sensitivity for further multi-component quantification and fingerprint analysis of GRG.

### 4.3 Applicability of QAMS for multi-component evaluation of GRG

The core of the QAMS approach is the establishment of stable RCFs between the IR and the other analytes. The selection principle was based on RSDs (generally, an RSD lower than 5% suggested a minor influence of error on RCFs) (Chen et al., 2022). According to the results in Figure 3A, the calculated RCFs were generally stable regardless of the compound selected as IR. Meanwhile, the results in Figures 3B–G suggested that the six components were in principle applicable as IRs. Among them, gastrodin was considered the most suitable IR because it is a major characteristic constituent of GR, is structurally stable, readily available as a reference standard, and is relatively economical (Luo et al., 2023; Xiao et al., 2023). Such suitability was further supported by the fingerprint profiling results. Gastrodin was consistently detected in all investigated batches, and the relative retention times of the characteristic peaks with respect to gastrodin showed very low variability across batches (RSD <1%), indicating stable chromatographic behavior under the established method conditions. The specificity chromatogram also showed that the gastrodin peak was well resolved from the blank and adjacent peaks. In addition, gastrodin has also been adopted as the internal reference or key reference basis in previous QAMS study on GR as well as in the current CNS (Li et al., 2019; National Medical Products Administration, 2025). Hence, gastrodin was chosen as the final IR for subsequent QAMS analysis. Using grastrodin as the IR, the RCF deviations were all below than 3%, indicating good reproducibility and supporting the applicability of the QAMS method for the simultaneous determination of multiple constituents in GRG (Chen et al., 2022; Zhang et al., 2019). The subsequent comparison between ESM and QAMS results also verified the feasibility of the established QAMS for GRG (Figures 4A–F).

According to the simultaneous quantification results of six target components in GRG (Figure 4G), although the major characteristic constituents of GRG were consistently detectable, their relative abundances varied among batches. Such differences may be associated with variation in raw material quality, processing, or manufacturing conditions. This further indicated that multi-component determination is necessary for GRG, as reliance on a single marker alone would not adequately reflect batch-to-batch compositional variation.

### 4.4 Chemical consistency and batch variation revealed by fingerprinting and chemometrics

Compared with single- or limited-marker quantification, fingerprint analysis captured broader compositional variation among batches, including differences in unidentified but potentially relevant constituents. The fingerprinting results in Figure 5 show that most investigated GRG batches shared a generally similar chemical profile, while several batches, particularly S5, S7, S10, and S14, displayed measurable compositional deviation. The batches from Guangdong Yifang Pharmaceutical Co., Ltd. generally exhibited high mutual similarity, with the exception of S10, suggesting relatively consistent chemical profiles among the sampled products from this manufacturer. However, given the limited number of manufacturers represented and the uneven distribution of samples, these findings do not support broad conclusions regarding overall market distribution or manufacturer-specific quality characteristics. Future studies should include a wider range of manufacturers and more batches per source to further evaluate the generalizability of the method and the robustness of inter-manufacturer comparisons.

Among the investigated samples, batch S5 showed the most pronounced overall reduction in TLC response, quantified marker content, and fingerprint peak intensity. This pattern suggests marked compositional deviation relative to the other batches and may reflect lower quality of the original herbal material and/or differences in extraction or manufacturing processes. However, because the analyzed material was a finished granule product and detailed product information such as DER native and excipient use was not uniformly available for all batches, the present data do not allow a definitive conclusion regarding the pharmacopoeial compliance of the original crude drug. Accordingly, the observations for S5 should be interpreted as evidence of significant chemical deviation within the investigated commercial sample set rather than as a formal regulatory judgment on product acceptability. Overall, this pattern suggests that HPLC fingerprinting adds information beyond targeted quantification by capturing broader variation across both identified and unidentified constituents.

To further explore batch-to-batch variation and sample clustering, chemometric analyses were performed. According to the results in Figure 6, most batches showed broadly similar chemical compositions, whereas S5, S7, S10, and S14 exhibited relatively greater chemical variability. The distinct behavior of several batches may reflect differences in raw material quality, extraction efficiency, or manufacturing conditions, although the present data do not allow these factors to be distinguished. The overall consistency between TLC, quantitative analysis, heatmap visualization, PCA, HCA, and radar plots supports the value of integrating multiple analytical dimensions in the evaluation of GRG quality.

### 4.5 Comparison between the DAC-aligned approach and the CNS

The current CNS provides an established basis for the quality assessment of GRG. However, as summarized in Table 3, notable differences exist between the CNS and the DAC-aligned analytical approach adopted in this study with respect to TLC intensity evaluation, the expression and emphasis of SST, chromatographic platform, content expression, and the depth of batch consistency assessment. These differences reflect distinct analytical emphases and regulatory practices rather than fundamentally different quality objectives (Table 3).

**TABLE 3:** Comparison between the current CNS and the DAC-aligned analytical approach adopted in this study for GRG.

Analytical aspect	Current CNS for GRG	DAC-aligned analytical approach in this study
TLC reference system	Uses both HRS and reference substance for identification	Uses HRS and reference substance, with additional DAC/EP-oriented emphasis on graded reference solutions
TLC intensity evaluation	Mainly based on prescribed comparison under CNS conditions	Includes primary and diluted reference solutions to assess band intensity and chromatographic response across concentration levels
TLC SST	Identification conditions are prescribed, but paired-marker SST is less explicitly emphasized	SST performed using two chemically defined markers (gastrodin and parishin B) to verify adequate chromatographic separation
HPLC platform	UHPLC with CNS-prescribed conditions	Conventional HPLC selected for broader applicability and transferability
HPLC SST	Explicitly requires theoretical plates calculated on gastrodin ≥5,000	Emphasizes chromatographic suitability based on resolution between structurally defined neighboring compounds (parishin B and parishin C)
Quantitative strategy	Includes QAMS-based multi-component determination in the CNS	Also uses QAMS, but as part of an integrated DAC-aligned strategy combined with explicit SST and fingerprint-based evaluation
Content expression	Combined six-constituent content expressed as a specified range (43.0–80.0 mg/g)	Total content of the six quantified constituents described in percentage form (>3.5% in most investigated batches) as a descriptive comparative observation
Batch consistency evaluation	Primarily standard-based identification and quantification	Additional HPLC fingerprinting plus chemometric analysis for multi-batch chemical consistency assessment

One major difference lies in the TLC identification strategy. In the DAC/EP-oriented approach, both a primary reference solution and a diluted secondary reference solution of gastrodin were used. This design enables assessment not only of band position, but also of chromatographic sensitivity, response intensity, and system performance across concentration levels. In addition, the TLC SST in the present study was based on the separation of two chemically defined markers, gastrodin and parishin B, thereby providing a direct assessment of chromatographic suitability. By comparison, the CNS procedure places less emphasis on such paired-marker system suitability evaluation. From the perspective of cross-standard assessment, the DAC-oriented approach therefore introduces a more explicit chromatographic suitability requirement for qualitative identification.

Differences are also observed in the chromatographic platform and the SST expression. The CNS adopts ultra-high-performance liquid chromatography (UHPLC) and explicitly requires that the number of theoretical plates, calculated on gastrodin, should not be less than 5,000. In contrast, the DAC-aligned approach established in this study employed a conventional HPLC system with a standard C18-AQ column, achieving adequate chromatographic separation with a resolution greater than 4.5 between parishin B and C in the SST. This result indicates that a DAC-aligned multi-component analytical strategy can be implemented using more widely accessible instrumentation while still meeting stringent chromatographic suitability expectations.

Further differences are evident in the expression of quantitative evaluation. In this study, six characteristic constituents were simultaneously quantified by QAMS, and most investigated batches showed a total content above 3.5%. This value is reported here as a descriptive observation derived from the present sample set. By comparison, the CNS specifies a content range of 43.0–80.0 mg/g for the combined six constituents. These results illustrate that different standard systems may use different formats and interpretive frameworks to describe similar quality attributes of GRG.

Overall, the comparison suggests that the CNS and DAC-oriented approaches are complementary in their assessment of GRG quality. The CNS provides an established and practical framework for routine national quality control, whereas the DAC-aligned strategy places greater emphasis on chromatographic suitability and multi-dimensional characterization. For herbal granule products intended for broader international use, such comparative evaluation may be particularly valuable for understanding how analytical expectations differ across compendial contexts.

## 5 Conclusion

This study established and evaluated a DAC-aligned analytical strategy for the quality assessment of GRG based on TLC identification, QAMS-based multi-component quantification, and HPLC fingerprinting. The TLC method was suitable for the qualitative identification of characteristic constituents, the QAMS method enabled reliable simultaneous determination of six marker compounds using gastrodin as the internal reference, and the HPLC fingerprint provided reproducible chemical profiling for assessment of batch-to-batch consistency. The combined results indicate that this integrated approach is feasible for the multi-dimensional quality evaluation of GRG.

In comparison with the current CNS, the DAC-aligned strategy placed greater emphasis on chromatographic suitability and comprehensive component characterization. This study therefore provides a practical reference for cross-standard comparison and for the harmonization of quality evaluation approaches for herbal granule products in different regulatory settings.

## Data Availability

The datasets generated and/or analyzed during the current study are not publicly available due to regulation confidentiality. Requests to access the datasets should be directed to the corresponding authors.
